# Insights into Barley Root Transcriptome under Mild Drought Stress with an Emphasis on Gene Expression Regulatory Mechanisms

**DOI:** 10.3390/ijms20246139

**Published:** 2019-12-05

**Authors:** Agnieszka Janiak, Miroslaw Kwasniewski, Marta Sowa, Anetta Kuczyńska, Krzysztof Mikołajczak, Piotr Ogrodowicz, Iwona Szarejko

**Affiliations:** 1 Institute of Biology, Biotechnology and Environmental Protection, Faculty of Natural Sciences, University of Silesia in Katowice, 40-032 Katowice, Poland; 2 Center of Bioinformatics and Data Analysis, Medical University in Białystok, 15-269 Białystok, Poland; 3 Institute of Plant Genetics, Polish Academy of Sciences, 60-479 Poznań, Poland

**Keywords:** barley, drought, roots, stress, transcription factors

## Abstract

Root systems play a pivotal role in coupling with drought stress, which is accompanied with a substantial transcriptome rebuilding in the root tissues. Here, we present the results of global gene expression profiling of roots of two barley genotypes with contrasting abilities to cope with drought that were subjected to a mild level of the stress. We concentrate our analysis on gene expression regulation processes, which allowed the identification of 88 genes from 39 families involved in transcriptional regulation in roots upon mild drought. They include 13 genes encoding transcription factors (TFs) from AP2 family represented by ERFs, DREB, or B3 domain-containing TFs, eight WRKYs, six NACs, five of the HD-domain, MYB or MYB-related, bHLH and bZIP TFs. Also, the representatives of C3H, CPP, GRAS, LOB-domain, TCP, Tiffy, Tubby, and NF-Ys TFs, among others were found to be regulated by the mild drought in barley roots. We found that drought tolerance is accompanied with a lower number of gene expression changes than the amount observed in a susceptible genotype. The better drought acclimation may be related to the activation of transcription factors involved in the maintenance of primary root growth and in the epigenetic control of chromatin and DNA methylation. In addition, our analysis pointed to fives TFs from ERF, LOB, NAC, WRKY and bHLH families that may be important in the mild but not the severe drought response of barley roots.

## 1. Introduction

Roots are the first organs that perceive the signal of drought stress. Limited water availability has to be sensed by roots, and then the signal is transmitted to the above-ground parts of the plant [1]. At the same time, the root system exhibits large developmental plasticity under abiotic stresses, and understanding the mechanisms that trigger these changes is important to select key factors that are responsible for better plant adaptation to the adverse environmental conditions [2]. The essential role of roots under the abiotic stresses is accompanied by the activation of transcriptome changes, which create a molecular network of responses, and some of its elements may improve plant tolerance to the stress. The spectrum of changes at the molecular level is very broad and includes the alteration of many metabolic pathways, the synthesis of osmoprotectants and antioxidants, or proteins involved in hormone signaling [3,4]. Any transcriptional change relies on the action of transcription factors (TFs) and other proteins that are responsible for gene expression regulation. Factors that are differentially expressed under drought stress belong to various TF families, including AP2/ERF, NAC, bZIP, MYB/MYC, bHLH, WRKY, CAMTA, Alfin-like, or homeodomain TFs [5,6] and represent both positive and negative regulators of drought response [7,8,9]. Specific members of these families have been used to construct overexpression lines that have proven their role in the increase of drought tolerance, either by changes in root growth and architecture [10,11], root anatomy [12], or biochemical composition of root cell walls [13]. The importance of gene expression regulatory mechanisms makes TF encoding genes one of the main targets in the efforts aimed at the development of new varieties better adapted to water scarcity conditions. For this reason, we perform a study of root transcriptomes under water deficit, which we focused mainly on the discovery of gene expression regulatory factors that are involved in the regulation of drought response in this organ. We used barley for this analysis, as it is one of the important cereal species encompassing around 20% of cereal production in Europe [14]. Also, the availability of the barley genome sequence and its annotation [15] makes it a good model to study gene regulatory network activated in response to drought.

Here, we analyzed two barley genotypes: a European cultivar “Maresi” (Mar) and a Syrian breeding line “Cam/B1/CI08887//CI05761” (CamB). Our previous studies have shown that CamB is more drought tolerant than the European cultivar. Its higher drought tolerance was noticed under severe stress conditions, when plants were subjected to 10 days of stress with the soil water content kept near the permanent wilting point [16,17], and also under mild stress of the same duration, which resembled more natural water shortage conditions [18,19]. Leaf and roots transcritome profiling of these genotypes under severe drought stress found genes that predispose the plant to better stress survival. They represent factors involved in the regulatory network of gene expression, signaling mechanisms, with significant contribution of hormone signaling pathways and an interplay between ABA, auxin, ethylene, and brassinosteroid homeostasis or genes encoding LIM domain proteins, which may function as osmotic biosensors. In the presented study, we performed a transcriptome analysis of the whole root system and the second leaf of CamB and Maresi genotypes subjected to 10 days of mild drought at the seedling stage. The transcriptomes of leaves served in the presented study as a background to depict which genes show expression changes in an organ-specific manner. Our data indicate that the mild drought results in more changes in the transcriptomes of roots than in the leaves, and more differentially expressed genes (DEGs) are root-specific. In roots of the CamB genotype, a similar number of DEGs were induced or repressed by the stress, whereas a down-regulation of gene expression prevailed in the roots of more drought susceptible Maresi. We identified 88 DEGs that encode transcription factors or gene expression regulators and the majority of them are root-specific. Their probable function in drought response and tolerance is further discussed, pointing to a possible regulatory network downstream of the selected transcription factors. 

## 2. Results

### 2.1. An Overview of Root and Leaf Transcriptome Changes in Response to Mild Drought Stress

Treatment of barley seedlings with a mild drought stress allowed the identification of 2087 differentially expressed genes. We observed a comparable number of DEGs in both genotypes. About 1000 DEGs were found in roots, and nearly 800 were observed in leaves of each genotype (Table 1 and Table S1). A similar number of DEGs were up- or down-regulated in roots of the drought-treated CamB genotype, whereas in the roots of Maresi, the number of down-regulated genes prevailed over the up-regulated ones. In leaves, more genes were up-regulated than down-regulated by the stress in both genotypes.

**Figure 1:**
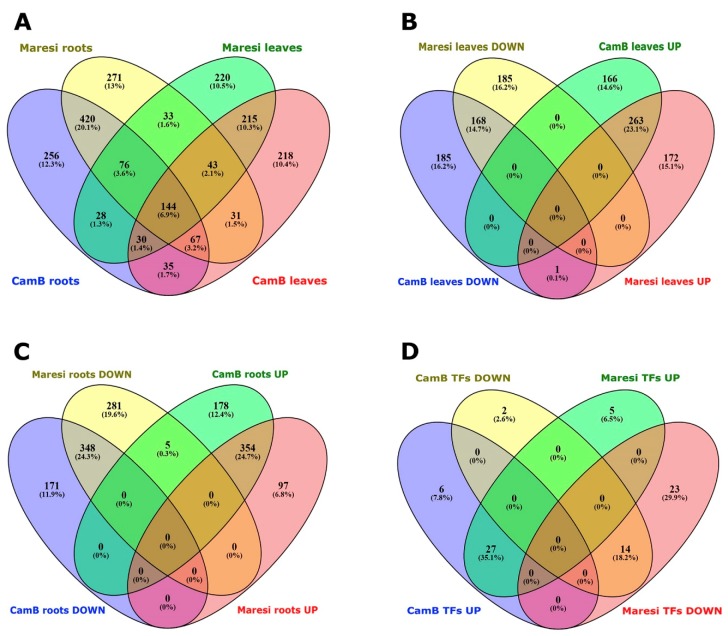
Venn diagrams representing several groups of differentially expressed genes (DEGs). (**A**) The comparison of a number of DEGs between roots and leaves of CamB and Maresi genotypes. (**B**) The comparison of a number of up- and down-regulated DEGs in leaves. (**C**) The comparison of a number of up- and down-regulated DEGs in roots. (**D**) The comparison of a number of up- and down-regulated DEGs encoding transcription factors and gene expression regulatory proteins identified in the roots of CamB and Maresi.

Hierarchical clustering of all samples showed that the organ (roots or leaves) was the most discriminative factor dividing the samples into two major transcription profile groups. Smaller differences in transcriptomes were related to the growth conditions (control vs. stress), and the genotype was the least discriminative (Figure S1). The results of hierarchical clustering was supplemented with the values of correlation coefficients between microarray data from each combination of genotype, organ and the type of treatment, calculated for all biological replicates (Table S2). When root and leaf transcriptomes were compared, we found that 947 DEGs were root-specific and much less, 653 DEGs, were expressed specifically in leaves. A group of 144 genes were differentially expressed in an organ- and genotype-independent manner and a set of 343 genes formed different combinations of organ-genotype specific expression patterns (Figure 1A). The whole set of identified DEGs was then functionally analyzed using the KEGG orthology assignment and BRITE functional hierarchies. Importantly, only 25% of barley DEGs were successfully assigned to KEGG orthology groups and were possible to be mapped into KEGG pathways (Table S3). The BRITE functional hierarchies of identified orthologous groups showed that the majority of DEGs encode enzymes of various metabolic pathways. The remaining genes encode proteins involved in cellular transport, signaling, photosynthesis, DNA repair, or macromolecules protection. We also found a subset of genes involved in gene expression regulation, including transcription factors, or mRNA, tRNA, and ribosome biogenesis (Figure S2). Then, we analyzed root and leaf transcriptomes separately, taking into account the direction of gene expression changes. In roots, the highest number of DEGs, altogether 702 genes, were either up- or down-regulated, commonly in both genotypes. Almost 350 DEGs were up- (178 DEGs) or down-regulated (171 DEGs) exclusively in roots of the CamB genotype, and 378 genes were differentially expressed exclusively in Maresi (97 up-regulated and 281 down-regulated DEGs; Figure 1B). Additionally, five genes showed opposite expression changes in both genotypes—down-regulation in Maresi and up-regulation in the CamB genotype. In leaves, we found a similar number of DEGs, around 350, that were present exclusively either in the CamB or Maresi genotypes. More DEGs were commonly up-regulated than down-regulated in both genotypes (Figure 1C), and one gene had the opposite expression pattern—it was down-regulated in CamB and up-regulated in leaves of the Maresi cultivar. 

In order to check the reliability of microarray data, ten randomly selected root DEGs were analyzed using the qPCR method (Table S4). In all cases, significant differences in the fold change of gene expression between control and drought treated samples were obtained. Spearman’s rank correlation coefficient (Rs) analysis showed a high correlation in a fold change of expression in both types of data (Rs = 0.856; *p* < 0.001; Figure S3), which indicates good quality of microarray analysis.

### 2.2. The Characteristics of Root Transcriptomes of Barley Genotypes Exposed to Mild Drought Stress

Due to a relatively small fraction of genes successfully assigned to KEGG orthology groups, we extended the functional analysis of DEGs detected in roots by gene ontology (GO) enrichment analysis (Table S5), which was later generalized using the GO slim approach. It showed that 10 days of mild drought stress resulted in a significant change of many processes important for root function. The highest number of DEGs belongs to various aspects of biosynthesis processes and the nucleobase-containing compound metabolism, including mostly the regulation of gene expression. Many genes take part in redox processes, cellular transport, protein modification, response to stress and the metabolism of various macromolecules (Figure S4A). Within the majority of these processes, a similar number of genes was either up- or down-regulated, with the exception of protein modifications and carbohydrate metabolism, where more genes were found to be down-regulated. Interestingly, several genes related to photosynthesis process were differentially expressed in roots of both genotypes subjected to drought stress. They represent five genes encoding subunits of photosystem I reaction center, two proteins from photosystem II, plastocyanin, two oxygen-evolving enhancer proteins, and the chlorophyll a-b binding protein. Remarkably, none of these genes were differentially expressed in leaves after the 10-days of stress, although we found other genes involved in photosynthesis to be differentially expressed in leaves (Table S6). 

Other biological processes that were mostly down-regulated in roots represented reproduction and pollen-pistil interactions, and the majority of genes that were grouped by this GO term encode proteins with serine/threonine kinase activity. The remaining processes affected by the stress were related to translation, signal transduction, cell communication, cell cycle, and cell growth, among others. 

The categorization of genes into groups by their molecular function allow us to point to two main categories: binding and several enzymatic activities, represented by oxidoreductases, transferases, hydrolases, or kinases (Figure S4B). Within the binding function, the majority of genes play a role in gene expression regulation, and within the genes encoding kinases, hydrolases, and transferases, many are important in cellular signaling pathways. 

The last aspect of gene ontology analysis, cellular localization of proteins encoded by the identified DEGs, showed that their highest number belongs to the membrane proteins, but the important fraction is also localized in the nucleus or extracellular region (Figure S4C). 

### 2.3. Root DEGs Involved in the Regulation of Gene Expression 

A relatively large group of root DEGs function in the regulation of gene expression. We identified 77 genes encoding various transcription factors belonging to 33 different families, which changed their expression upon the stress in roots. Among these DEGs, eight were differentially expressed specifically in roots of the CamB genotype and 27 were specific to Maresi cultivar, whereas 27 and 14 DEGs were respectively up- or down-regulated in roots of both genotypes (Figure 1D). 

**Figure 2:**
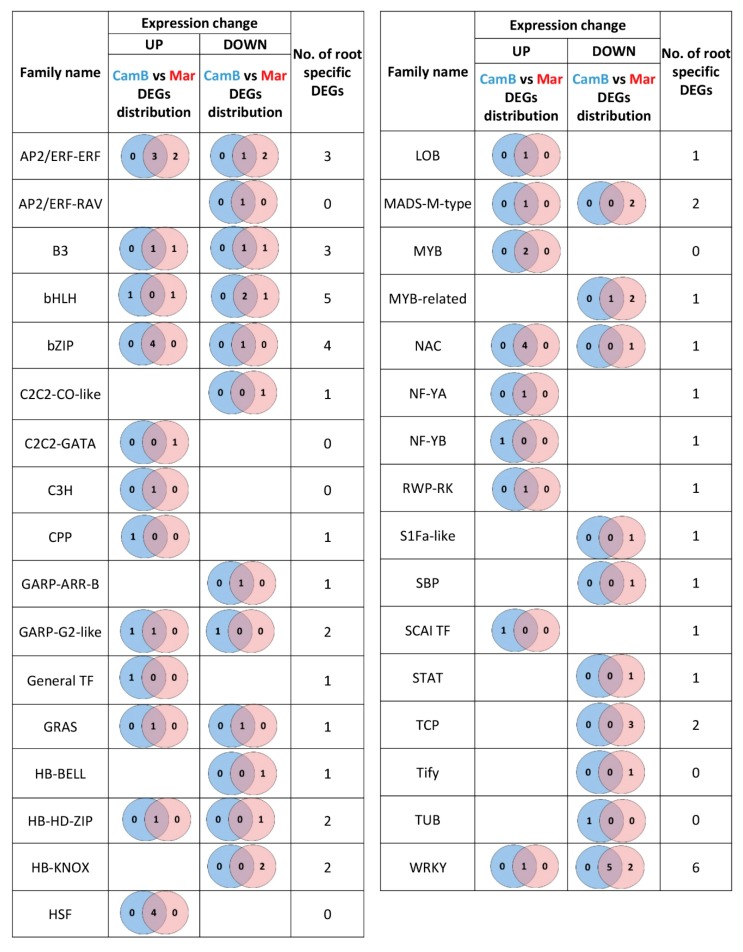
An overview of the gene number and root-specific differentially expressed genes (DEGs) encoding transcription factors differentially expressed after mild drought treatment in roots of CamB and Maresi genotypes. Cam—CamB genotype, Mar—Maresi genotype.

About 60% of all these DEGs were differentially expressed only in roots of at least one genotype, while the remaining genes were also found as DEGs in leaves of either CamB or Maresi (Figure 2, Table S7). The root-specific DEGs encoding TFs belong to 15 different families, and in the majority of cases, these families comprised of one DEG only. The exception is a bHLH TF family that consists of five DEGs and the families that possess a homeodomain coupled with other functional domains, where the other five genes were classified (HB-BELL, HB-HD-ZIP, and HB-KNOX families). An overrepresentation of root-specific DEGs was also found in the bZIP and WRKY families (Figure 2, Table S7). 

In addition to the group of DEGs for transcription factors, eleven DEGs were classified as genes encoding other types of transcriptional regulators. They include auxine responsive proteins of the AUX/IAA family, mitochondrial transcription termination factors (mTERF), the PHD finger proteins, tumor necrosis receptor-associated factor (TRAF) family proteins, an ethylene receptor, and a protein with BAH domain. Eight of these DEGs were down-regulated, and nine were root-specific (Table S7). 

In order to find the possible regulatory factors that may contribute to the drought tolerance or drought susceptibility, we selected the genes that were differentially expressed in roots in a genotype-specific manner. There were altogether ten DEGs specific to the drought-tolerant CamB genotype, representing eight genes encoding transcription factors—six of them were up-regulated, and seven were differentially expressed only in the roots of this genotype. There were also two genes encoding regulatory proteins, one up- and one down-regulated, and both of them were specific to the roots (Table 2). 

A similar analysis was performed for the drought susceptible cultivar Maresi, and here, almost 3× more DEGs (28 DEGs) encoding transcription factors were found to be specific to this genotype. The majority (23 DEGs) were down-regulated, and the expression of most of them was not affected by the stress in leaves. Among those genes that were differentially expressed also in Maresi leaves, one gene, encoding a protein from the TIFY family, showed an opposite expression change in the two organs. Additionally, three genes encoding transcriptional regulators were also found. Two of them were specific to the roots of Maresi, and one showed an opposite expression pattern in leaves and roots. This gene encodes a putative E3 ubiquitin-protein ligase and was also up-regulated in the leaves of the CamB genotype (Table 3).

### 2.4. Target Genes Putatively Regulated by Specific Transcription Factors with Differential Expression in Roots

To depict some possible regulatory connections between specific transcription factors and their putative target genes, we have subjected all genes differentially expressed in roots of both genotypes to the analysis in the Transcription Factor Enrichment tool (http://plantregmap.cbi.pku.edu.cn) that allows us to find the TFs which possess significantly over-represented targets in the input gene list. For this purpose, the whole list of root DEGs was divided according to the direction of their expression change, and then the two sets of genes were used as queries against the TF enrichment tool. It was possible to find four TF genes that were differentially expressed after the mild drought stress in roots and possessed overrepresented targets in our data set (Table 4 and Table S8). Three of them were up-regulated, and one (MLOC_81003, encoding a TF from bZIP family) was down-regulated by the stress in both genotypes. All their putative target genes had the same direction of expression change—up-regulation, which indicates that the MLOC_81003 gene may act as a negative regulator of these putative targets. 

**Figure 3:**
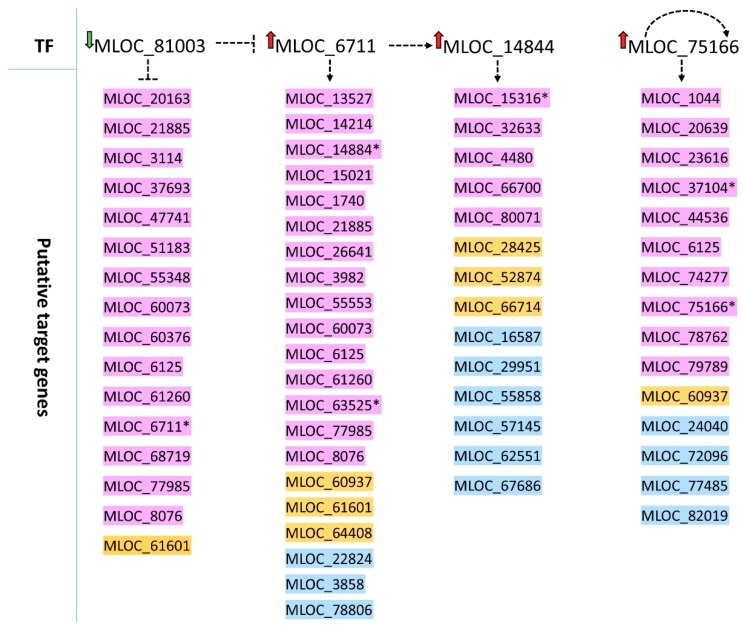
Putative targets and predicted regulatory network of these TFs, which possess significantly over-represented targets within root DEGs identified after mild drought stress. Violet—DEGs common to CamB and Mar genotypes; orange—DEGs specific to Maresi; blue—DEGs specific to CamB. Asterisk—DEGs encoding TFs.

We also found that some regulatory cascades may exist between the identified TF genes. The MLOC_14844 encoding a TF from Myb-like family was within the targets of MLOC_6711, a gene for a G-box binding factor 2, belonging to the bZIP family, indicating a possible regulatory connection between these two genes. The latter may be under a negative regulation of MLOC_81003 (another bZIP TF), and a MLOC_75166 encoding heat stress TF may be auto-regulated as this gene was found among its own targets (Figure 3, Table S8). This regulatory network may be further extended based on other identified target genes. The analysis showed that MLOC_14844 may regulate the expression of another G-box binding transcription factor (MLOC_15316). The MLOC_6711 may be responsible for the activation of a gene from C3H family (MLOC_63525), and MLOC_75166 may regulate another two TF-encoding genes representing NAC family (MLOC_37104 and MLOC_23616), The last one was annotated as a protein with unknown function, but it possesses domains characteristic to NACs (Table S8). 

The function of the putative targets for the four TFs spans several categories, such as metabolism, including nucleic acid metabolism, gene expression regulation with the above-mentioned transcription factors, and other molecules involved in transcription regulation, translation initiation, or ribosomal proteins. Other genes belong to stress response signaling and redox processes, transmembrane transport or plastid formation. We have not found any specific preferences of the four TFs to target genes from a defined biological process; they may all regulate expression of genes of the broad spectrum of biological functions. 

### 2.5. A Comparison of DEGs Involved in Gene Expression Regulation in Roots during Mild and Severe Drought Stress

The drought response on the transcriptional level of the same barley genotypes (CamB and Maresi) was previously analyzed under severe drought stress, where the seedlings at the same stage as presented here were grown for 10 days in the soil moisture close to the point of permanent plant wilting [20]. Thus, it was possible to check which genes involved in gene expression regulation in roots are common or specific to the different drought regimes. 

**Figure 4:**
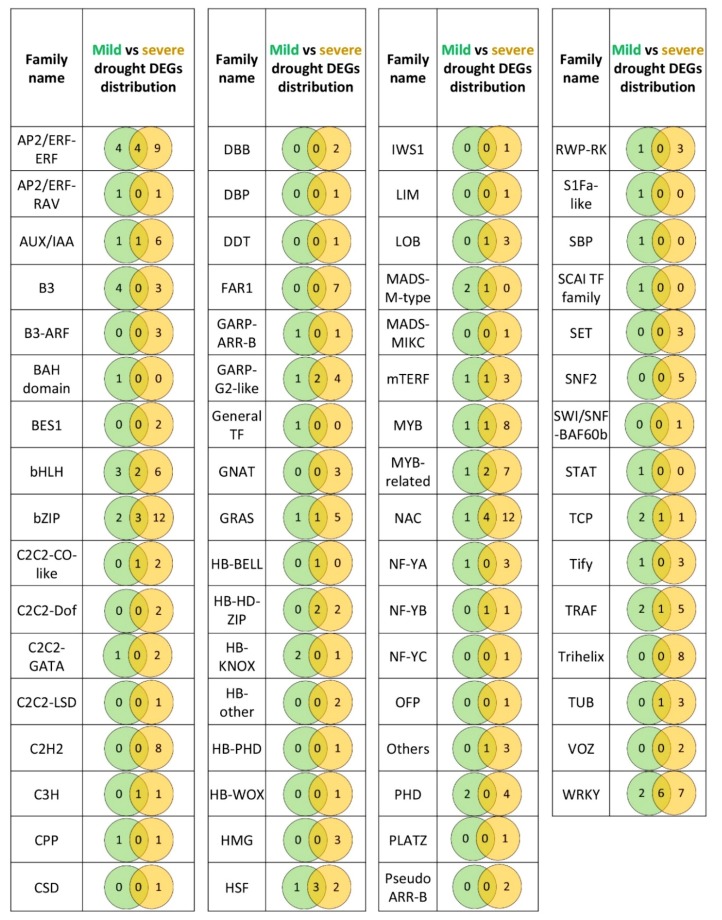
An overview of the number of TFs genes differentially expressed in roots after mild and severe drought treatment.

Altogether, the severe drought stress resulted in the expression changes of 187 DEGs encoding transcription factors that belong to 47 different families and 43 DEGs encoding other proteins involved in transcription regulation from 12 different families. Of this number, 42 DEGs were common for severe and mild drought stress, including 38 DEGs for transcription factors and 4 DEGs for other regulators of gene expression. Mild drought-specific DEGs included 47 genes, and 40 of them encode transcription factors. The remaining 188 DEGs were specific to the severe drought stress (149 genes for TFs and 39 genes for other types of expression regulators; Figure 4, Table S9). 

Factors from AP2/ERF family, B3, and bHLH families predominated within the group of DEGs specific to mild drought (Figure 4). Moreover, genes from MADS-M-type, S1Fa-like, SBP, SCAI, and STAT families were differentially expressed after the mild drought stress only. On the other hand, the severe drought stress resulted in a specific differential expression of genes from 20 TF families and the most numerous were the C2H2, Far1, and Trihelix families. Many DEGs specific to severe drought also belong to the AP2/ERF, bZIP, bHLH, NAC, and WRKY families. 

**Figure 5:**
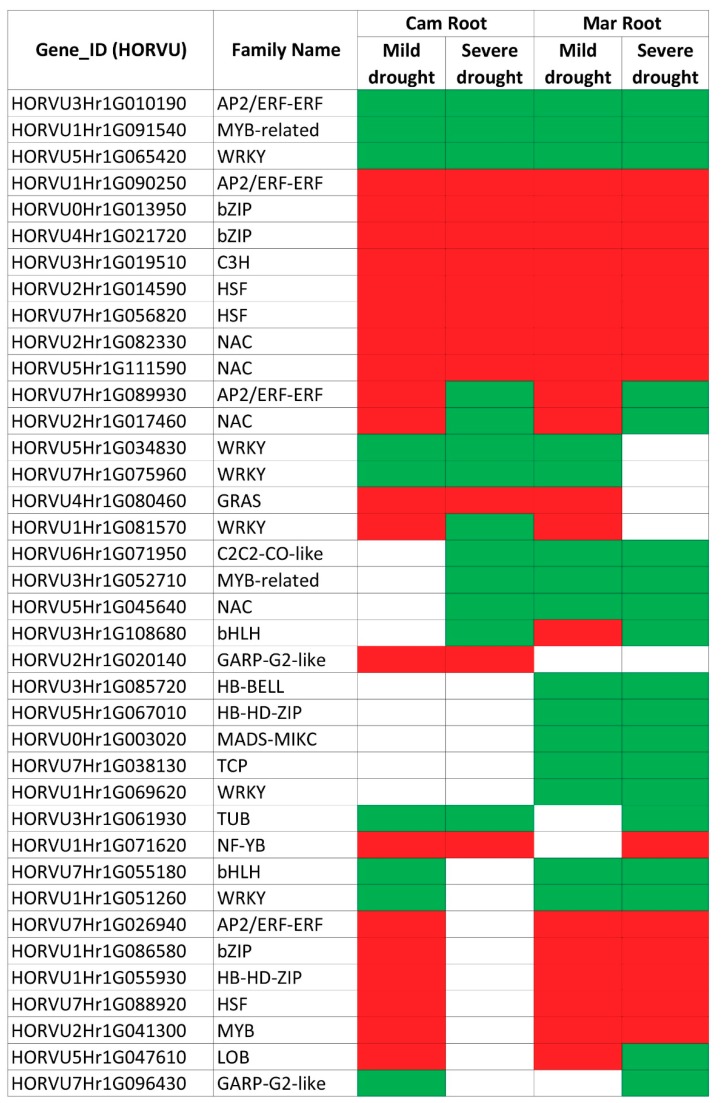
The expression pattern of DEGs encoding transcription factors, which were differentially expressed in roots of barley seedlings subjected to mild and severe drought regimes. Green: down-regulation of a gene; red: up-regulation of a gene.

Within DEGs common to both stresses, again, factors from AP2/ERF, bZIP, bHLH families were found, together with genes belonging to the heat shock, NAC and WRKY families, among others. Importantly, the pattern of gene expression changes after mild and severe stress or between the two genotypes was not uniform for all DEGs (Figure 5, Table S10) and interestingly, we found some DEGs that showed opposite expression change in both drought regimes: up-regulation after mild and down-regulation after severe drought (Table 5). All of these DEGs, but one, were specific to roots, they did not change their expression in leaves in either of drought regimes.

## 3. Discussion

Our analysis of root transcriptomes of two barley genotypes, which differed in their level of drought tolerance, allowed us to select several genes encoding transcription factors that may be involved in better drought survival. Among the ten genes that were specifically activated or repressed in roots of drought-tolerant CamB, one encodes the TSO1-like CXC protein, which is the homolog of the human TESMIN gene that is expressed mainly in testes and ovaries [21]. In Arabidopsis, the TSO1 gene functions in floral tissue development, inflorescence meristem organization, and control of cell division [22]. Importantly, it is required for the correct organization of shoot and root apical meristems [23]. It was shown that TSO1 acts as a repressor of the *MYB3R1* gene, which in turn maintains the repressed state of G2/M-specific genes in the roots, preventing cell proliferation. Thus, high expression of *TSO1* allows the maintenance of root proliferation in the root meristematic zone [23]. In our study, the up-regulation of the *TSO1-like* gene in CamB roots suggests that it may be involved in the promotion of better drought tolerance of this genotype. 

In our data set, no differential expression of the *MYB3R1* homologue was noticed, but we found two other genes from the MYB superfamily, belonging to the GARP-G2-like group that were specific to CamB roots (HORVU2Hr1G020140 and HORVU7Hr1G096430). The first one was up-regulated and was annotated as two-component response regulator *ARR18*, but the homology search in Ensembl Plants database showed that its orthologues in Arabidopsis (*HRS1*, *HHO2*, or *HHO3*) and rice (*NIGT1*) are involved in nitrate and phosphate signaling in roots [24] and in the presence of nitrate they repress the nitrate-starvation response [25]. The second one was down-regulated in CamB roots and is the orthologue of Arabidopsis *PHL6* and rice *PHR4* genes, that are activated upon limiting phosphate availability and regulate phosphate-starvation response pathway [26]. It was shown that high nitrate reduces lateral root elongation [27] and a similar observation was true for drought treated plants [28]. On the other hand, phosphorus starvation increases lateral root formation and the density of root hairs [27], placing these genes in a pathway that may influence the root plasticity in response to the soil environment. Expression pattern of barley orthologues of NIGH1 and PHR4 observed in our study suggests that they may also play a role in drought response although their precise function in this type of stress remains to be elucidated. 

An interesting gene (HORVU5Hr1G062040) that was up-regulated in CamB roots shows similarity to human *SCAI*, the suppressor of cancer cell invasion gene that inhibits the invasive migration of tumor cells [29]. SCAI protein interacts with KDM3B, a histone demethylase protein that removes methyl groups from H3K9me1/2, which is the mark of repressed chromatin state [30], and with the SWI/SNF chromatin remodeling complex [31]. Thus, the HORVU5Hr1G062040 gene plays a role in the regulation of chromatin status and its transcriptional availability. Two other DEGs involved in epigenetic regulation were found in CamB roots: a down-regulated HORVU5Hr1G061120 gene encoding a bromo-adjacent homology (BAH) domain-containing protein and an up-regulated HORVU6Hr1G034680 gene for a methyl-CpG-binding domain-containing protein. The proteins with a BAH domain recognize methylated state of histone H3 lysines that are the hallmarks of the heterochromatin [32]. The methyl-CpG-binding proteins interact with methylated CpG sites in DNA and are also responsible for silencing the target chromatin regions [33]. The direction of expression changes of these genes in the roots of the CamB genotype suggests that the efficient response to drought may require the formation of an active chromatin status of genes that are targeted by SCAI and BAH proteins. The repression of transcription of other genes, which are under the regulation mediated by the methyl-CpG-binding protein, may be also needed. 

In the roots of Maresi, contrary to CamB, more genotype-specific DEGs for gene expression regulatory proteins were found, and the great majority of them were down-regulated by the stress. This finding suggests that in a drought-susceptible cultivar, more dynamic changes occur after the drought treatment. It is not clear whether the prevalence of down-regulatory mechanisms is an indicator of a lower acclimation ability to the stress or it just suggests that Maresi enters a substantially different pathway of drought response than CamB. Many detected TFs belong to large families with members of positive and negative expression regulatory functions, and there are reports of their both up- or down-regulation upon drought. Such observations were made, for example, for genes from AP2/ERF [34,35], bHLH, MADS, MYB [8], NAC [9], or WRKY families [7]. Thus, a more detailed analysis targeted to single genes is needed to detect their specific impact on drought response in the Maresi cultivar. Nevertheless, some possible molecular response pathways have emerged that may be characteristic of the roots of this drought-susceptible genotype. 

One pathway may be connected to jasmonic acid (JA) signaling and the action of three genes: HORVU7Hr1G041230, HORVU3Hr1G050590, and HORVU4Hr1G003040 encoding a TIFY3A TF, WRKY and an NPR1 protein, respectively. The TIFY3A TF belongs to the JAZ subfamily of TIFY, which is known to repress the action of Arabidopsis MYC2 TF and its homologues—the positive regulators of JA-dependent responses. The important effect of such response is an inhibition of primary root growth [36]. The involvement of TIFY TFs in drought tolerance was demonstrated by the overexpression study of rice *OsTIFY11a*, which resulted in the enhancement of dehydration stress resistance [37]. Down-regulation of this gene in Maresi roots suggests that this pathway of possible drought tolerance may be negatively affected in this cultivar. 

Similar, negative regulation of JA response was found in Arabidopsis for another factor, *WRKY50* [38], a homologue of which HORVU3Hr1G050590 was again down-regulated in roots of Maresi. Another down-regulated gene (HORVU4Hr1G003040), encoding a regulatory protein NPR1, may also be placed within the JA-related response. It is a repressor of JA signaling that acts through the induction of the salicylic acid pathway [39], and its overexpression in Arabidopsis leads to oxidative stress resistance [40]. Taking all of this together, down-regulation of the above-mentioned genes may lead to the lack or lower rate of JA signaling repression in Maresi roots, which in turn may diminish the efficient drought response. 

Nonetheless, more complexity to the image of this response is added by the down-regulation of two *KNOTTED1-like* (*KN1*) transcription factor genes (HORVU2Hr1G061320 and HORVU5Hr1G098570). The KN1 TF negatively modulates the accumulation of gibberellin (GA) through the control of ga2ox1, which is an enzyme that leads to the catalysis of the GA [41]. Thus, down-regulation of *KN1* genes in Maresi roots may slow-down the degradation of GA, which in turn may promote root cell proliferation and elongation [42]. Moreover, it was shown that there is a crosstalk between GA and JA, mediated by MYC and DELLA proteins that compete with each other to bind with JAZ TF. In the presence of GA, DELLA protein is directed to degradation, and then JAZ and MYC may interact with each other, which prevents MYC from activation of JA-dependent genes and leads to the repression of JA-driven response [43]. This complex response of Maresi indicates the necessity of a substantial rebuilding of root metabolism under the mild drought in a susceptible cultivar, which does not take place in a tolerant genotype. 

Based on the enrichment analysis, we performed a prediction of possible relationships between differentially expressed TF genes and all DEGs that were identified in the roots of both genotypes. We found four TFs that had overrepresented targets among root DEGs, which may place them near the top of the regulatory network of the mild drought response in roots. Two of them encode TFs from the bZIP family: MLOC_81003 (HORVU5Hr1G014170) with the highest similarity to rice bZIP88 and MLOC_6711 (HORVU1Hr1G090030), similar to Arabidopsis G-box binding factor 2 (GBF2). Many bZIP TFs are involved in stress signaling and act in an ABA-dependent manner [44]. Our data suggest a possible negative regulation of *GBF2* by bZIP88 (HORVU5Hr1G014170) in drought response in barley roots, as the up-regulated *GBF2* gene was found among targets of down-regulated bZIP88. A down-regulation of *bZIP88* was also observed in rice subjected to various abiotic stresses, including drought [45]. Interestingly, a DNA binding activity of some G-group bZIPs was found to be regulated by light and reactive oxygen species (ROS) [46], which are commonly produced in tissues upon the abiotic stresses. Thus, one may hypothesize that ROS produced in drought treated roots may trigger the regulation of gene expression downstream of *GBF2*. Another TF gene with overrepresented targets encodes heat stress factor (HORVU7Hr1G056820) orthologous to rice, HSFB-2b gene. HSFs from class B are considered to be transcriptional repressors [47], contrary to our enrichments analysis data, which pointed to several up-regulated DEGs downstream to the *HFSB2b* gene (also up-regulated). On the other hand, a study of seed germination under heat stress in Arabidopsis showed the induction of the *HSFB2b* gene, which was accompanied by the accumulation of 49 heat shock proteins [48] (HSP). In our analysis, two up-regulated genes (HORVU3Hr1G007500 and HORVU3Hr1G020520) encoding HSPs were found as targets of HSFB2b. Moreover, a study of maize transcriptome suggests that the *HSFB2b* gene may be important in drought tolerance in this species, as *HSFB2b* expression was specifically activated in a drought-tolerant genotype [49]. Nevertheless, at this stage of our analysis, we treat the above enrichment data only as putative interactions that may serve to drive hypothesis on novel regulatory connections in the gene network, and which need to be verified based on gene-specific studies. 

The comparison of mild and severe drought stress regimes showed a significant increase in the number of DEGs involved in gene expression regulation when a severe drought was applied. Among all the regulatory DEGs detected after mild drought treatment, about 50% were also differentially expressed after severe stress. Additionally, the number of DEGs that were specific to only one stress regime was four times higher after severe stress, compared to the mild drought. Such results suggest that a substantial part of root drought response that is initiated upon moderate stress may also persist in more severe conditions. It is important to emphasize, however, that the details of this response differed in a genotype-dependent manner. A general scheme that emerged from our analysis suggests that the drought-sensitive genotype goes through more profound changes in the transcriptome than the drought-tolerant form, especially after the severe stress. The differences between the two drought regimes that were observed for DEGs with expression regulatory function were also reflected in the expression of all remaining genes involved in the other biological processes. Gene ontology analysis showed that DEGs identified after mild drought belong to similar biological and functional categories as genes differentially expressed after severe stress [20]. However, when we take into consideration the number of DEGs, then more DEGs are found within each GO category when the stress is stronger, and again, more DEGs are present in the drought-sensitive genotype [20]. The same was true for the TF DEGs detected in the presented study—a much higher number of TF DEGs were found after severe stress in each TF family, and this increase in number was more prominent in the Maresi genotype. A remarkably higher number of DEGs characteristic for severe drought were noticed, for example, within the AP/ERF family of ethylene responsive factors and AUX/IAA family involved in auxin signaling. A similar increase in gene number was noticed for the bZIP family, including factors involved in ABA signaling. It was shown that both hormones enter a crosstalk important for a drought response and trigger the inhibition of lateral root formation [50], making their regulatory network essential for shaping the root system architecture under stress. Severe stress also resulted in a higher number of genes from GRAS family, which may be placed within gibberellin signaling [51] and the genes from the Tify family involved in jasmonate signaling [52]. Interestingly, the Tify TFs detected under severe stress included down-regulated genes in CamB, suggesting that the drought-tolerant genotype may enter a similar response pathway related to jasmonates as the sensitive cultivar, but only after much more adverse environmental conditions. 

An interesting finding was the detection of five genes, which exhibited opposite expression changes after mild and severe stresses, and in the case of two of them, some functional characteristics may be related to the root function under drought. The first one (HORVU7Hr1G089930), which encodes a TF from the AP/ERF family is an orthologue of the Arabidopsis gene for the SHINE factor that is involved in wax synthesis. Overexpression of this gene caused the increase of wax biosynthesis and altered its composition, resulting in a higher cuticle permeability in leaves and better drought tolerance [53]. The Arabiopsis *SHINE* gene is also expressed in roots and is probably involved in suberin deposition [54]. Suberisation is considered to have a negative impact on water and solute transport, but it may also prevent an uncontrolled backflow of water to the surrounding soil under unfavorable environmental conditions [55]. During the day and in optimal soil moisture, the main force driving water transport is the transpiration, and water is transported via the apoplast. During the night or when stomata are closed due to drought stress, water flow from cell-to-cell and is driven by the root pressure. In the latter scenario, the suberin acts as a barrier limiting apoplastic transport [56]. The barley *SHINE* orthologue was up-regulated under mild drought in our study, but down-regulated after the severe water shortage conditions in both genotypes. It is possible that SHINE allows the increase of suberization when the signal of water shortage is perceived by the roots, and consequently, it helps to actively maintain the transport of limited, but still available water to the shoot, even if the transpiration rate is reduced. On the other hand, under the severe drought, when water is very limited, a high deposition of suberin may be unfavorable as it may increase hydraulic resistance of the root to the extent it prevents water acquisition. 

The second gene with the opposite expression change under moderate and severe drought (HORVU5Hr1G047610) belongs to the LOB (LATERAL ORGAN BOUNDARIES) family and was also up-regulated after mild drought in both genotypes and down-regulated in severe drought, but only in the Maresi cultivar. LOB transcription factors are involved in the development of lateral organs. The first LOB gene identified in Arabidopsis is expressed at the boundaries between stem cells and developing leaf primordia at shoot apical meristem [57]. Several *LOB* genes are important for lateral root formation driven by auxin signaling and modulated by ABA under stress conditions [58,59]. A maize *RTCS* gene from this family is responsible for the initiation and maintenance of embryonic seminal roots and shoot-born root primordia [60], and the *ARL1* gene in rice is involved in adventitious roots formation [61]. At present, it is difficult to predict the precise function of the *LOB* gene identified in the presented study in barley, but its differential expression in roots only and not in leaves suggests its importance for shaping root architecture and building an appropriate root system under the moderate but not in the severe stress conditions. 

Another aspect that emerged from our transcriptomic study was the discovery of genes that function in the photosynthesis process, but were differentially expressed under drought in roots of one or both barley genotypes. They included genes encoding proteins of oxygen-evolving complex, together with proteins that build photosystem I (PSI) and PSII in leaf chloroplasts, and most of them were up-regulated by drought stress. This finding is in agreement with our previous study, where several genes annotated as photosynthesis-related were also differentially expressed in roots upon severe drought [20]. We hypothesize that such result may suggest a specific role of root plastids as ROS scavenging centers, which may help root cells to cope with oxidative stress conditions generated by drought. Differential expression of genes involved in photosynthesis in roots under drought is not uncommon and was also detected in other studies, for example, in cotton [62], chickpea [63] poplar [64], and pine [65]. 

Our analysis also suggests that under the mild drought stress, different genes involved in photosynthesis are differentially expressed in leaves and the roots. We found, for example, up-regulation of a gene for RuBisCO activase A in Maresi leaves and differential expression of two genes encoding chlorophyll a-b binding proteins in leaves of both genotypes, whereas another gene for chlorophyll a-b binding protein was up-regulated in roots only. Similarly, we noticed down-regulation of a gene encoding one of PSII components in leaves, whereas, in roots, two other genes for PSII proteins were up-regulated. This may suggest that drought results in an organ-specific expression of genes with similar function, although we are aware that we analyzed only one time-point of the stress treatment, so we only spotted a subset of the dynamic changes of the transcriptomes and we cannot rule out that some of these genes exhibit different expression patterns at the early stage of the stress. Such possibility is partially supported by the research of Chmielewska et al. [18] who used plant material from the same experiment (the same genotypes and drought treatment) for the analysis of leaves and roots proteomes and metabolomes. On the protein level, they found a higher accumulation of oxygen-evolving enhancer protein in leaves of Maresi and a ferredoxin-NADP+ oxidoreductase in leaves of CamB, while in our study the differential expression of their transcripts was not found. It is likely that the up-regulation of transcription could occur at an earlier stage of the stress application and on the 10th day, the differences were visible only on the protein level. Similarly, Chmielewska et al. [18] found a higher accumulation of RuBisCO activase B in both genotypes and RuBisCO activase A protein in the leaves of Maresi only, while in our analysis, only the latter protein is in agreement with its transcript up-regulation. Nevertheless, taking together the above aspects of our analysis, the photosynthesis-related genes may be considered as a source of better drought response of roots, due to their possible anti-oxidative role protecting macromolecules from oxidative damage in this organ. 

To conclude, we have found a substantial number of transcription factor encoding genes that are involved in the drought response in roots of barley. The use of microarrays, despite their limitations, allowed us to make an unbiased comparison of transcriptome changes induced by mild drought to the response of the same genotypes to severe stress, which we studied previously [20]. The comparability of both analyses was ensured by the use of the same type of arrays and experimental methodology, except for the drought regime, in both experiments. We are aware, however, that additional use of the RNA-Seq method may give a more in-depth view on global gene expression changes. Nevertheless, our analysis suggests that genotype-dependent drought tolerance is accompanied by a lower number of gene expression changes than response of the susceptible genotype. The gene expression regulation is probably directed toward the maintenance of root meristems proliferation and a yet unidentified network of epigenetic changes that may allow a better acclimation of the plant to the stress conditions and may be triggered by factors involved in chromatin remodeling and DNA methylation. We found that a part of the drought response mechanisms is controlled by transcription regulators from Tiffy, WRKY, HB-KNOX, and mTERF families that may act within jasmonate and gibberellin signaling pathways. Our data also show that the activation of several TFs may be important in mild drought but not in severe drought response. We believe that the TF-encoding genes identified in the present study may be used as future targets for the enhancement of drought tolerance in barley and related species. 

## 4. Materials and Methods 

### 4.1. Plant Material and STRESS Treatment

Two barley genotypes were used for the study: a European semidwarf cultivar Maresi and a Syrian breeding line Cam/B1/CI08887//CI05761 (CamB), adapted to dry environments. Grains of both genotypes were sterilized, and initially, 20 grains were placed in the double-walled Kick–Brauckman’s pots of 10 dm^3^ capacity. After germination, the number of plants was reduced to 10. The pots were filled with a mixture of sandy loam and sand (7:2, *w*/*w*). The analysis of the water retention curve of this substrate showed that a pF range of 2.2–3.0 indicate easily available water. At the pF 3.0–4.2, water was less available, creating the conditions of drought stress, and pF > 4.2 was a permanent wilting point [19]. In the present study, two water regimes were used: control conditions with the pF range of pF 2.2–3.0 and a mild drought with pF value kept at the range of 3.4–3.6, which was applied at the 3-leaf seedling stage and lasted for 10 days. The experiment was started in spring (April), and plants were grown in a glasshouse with natural light conditions. The soil moisture was controlled daily using an FOM/mts TDR soil moisture meter according to the reflectometry method (Easy Test, Institute of Agrophysics PAS, Lublin, Poland) and adjusted by adding the appropriate quantity of water. The experiment was carried out in three replicates and tissue for RNA extraction was collected from five plants per replicate. 

### 4.2. RNA Isolation

After 10 days of drought stress, the second leaf and the whole root system were collected for RNA extraction from both drought-treated and control plants. To collect roots, plants were gently removed from the soil, separated from each other and roots were briefly washed in water to remove the soil substrate. The washing time did not exceed 30 s. Leaves and roots were frozen in liquid nitrogen and were subsequently homogenized in a sterile, ice-cold mortar. Total RNA was extracted using an RNeasy Plant Mini kit (Qiagen, Hilden, Germany), according to the manufacturer’s instructions. Extracted RNA was additionally purified using precipitation in 1 M lithium chloride, and each RNA precipitate was then dissolved in 15 μL of nuclease-free H_2_O. The yield and purity of the RNA were determined using a NanoDrop ND-1000 spectrophotometer (NanoDrop Technologies, Wilmington, DE, USA). The integrity of the RNA was checked using denaturation agarose gel electrophoresis using pre-cast gels and FlashGel RNA System (Lonza, Basel, Switzerland). 

### 4.3. Preparation of Microarrays and Microarray Data Analysis

The synthesis, labeling, and hybridization of cDNA and cRNA to 4 × 44K Agilent Barley gene expression arrays (Agilent Technologies, Santa Clara, CA, USA) were carried out at the Genomics Core Facility, European Molecular Biology Laboratory (EMBL), Heidelberg, Germany, as described earlier in Kwasniewski et al. [66]. One technical replication of microarray hybridization was made for each of the biological replications representing different experimental combinations of genotype, organs, and the type of treatment. The microarray data were analyzed using GeneSpring GX 12.5 software (Agilent Technologies). Hybridization data were subjected to per chip normalization using the percentile shift method to the 75th percentile. A baseline transformation was then performed to the median of all of the samples. Statistical testing for differential expression was performed using two-way ANOVA followed by the Benjamini–Hochberg false discovery rate (FDR) correction for multiple testing [67]. Fold change (FC) ≥3 (*p* ≤ 0.05 after FDR correction) was considered as a differential expression of a gene between drought-treated and control samples. Raw microarray data, normalized intensity values, and corresponding metadata are accessible through the Gene Expression Omnibus (GEO) repository under the accession number GSE128048.

### 4.4. Agilent Barley Gene Expression Microarray Annotation, GO Enrichment and Transcription Factor Encoding Genes Analysis

Blast2Go software (BioBam Bioinformatics S.L, Valencia, Spain) [68] and the blastn algorithm were used to map probes sequences from the Agilent Barley gene expression microarray to cDNA sequences representing high-confidence (HC) barley genes which were retrieved from the Ensembl Plants database (http://plants.ensembl.org), using the barley genome assembly version Hv_IBSC_PGSB_v2. The threshold values for the blastn settings were as follows: E-value—1.03 × 10^−3^, word size—9, and high-scoring segment pair (HSP)—33, with low complexity filter applied. The functional annotations of HC genes was used after Mascher et al. [15]. Because several online databases still utilize the data from the first version of barley genome assembly, similar annotation of microarray probes was done using the old version of the genome (version 082214v1, EnsemblPlants database). For this reason, all results in supplementary materials and, when applicable, in the main text, are presented using two gene identifiers abbreviated as HORVU (for the new genome assembly) and MLOC (for the old assembly). 

The functional characterization of all identified DEGs was performed using the KEGG database. To assign DEGs into KEGG orthologous groups, a BlastKOALA tool was applied [69], and the KEGG identifiers were then used to reconstruct KEGG pathways and BRITE hierarchies available at KEGG service (https://www.genome.jp/kegg/kegg2.html). Gene ontology enrichment analysis was carried out separately for up- and down-regulated root DEGs using singular enrichment analysis (SEA) available through the AgriGO v2.0 toolkit (http://systemsbiology.cau.edu.cn/agriGOv2). Here, a hypergeometric statistical test, at a significance level of *p* = 0.01 and minimum mapping entries of 5, was used. The GO data for the whole set of barley genes from the IPK Barley BLAST server were taken as a background reference for the analysis. The individual enrichment results were then compared using the SEACOMPARE tool from AgriGO v2.0. The GO slim analysis was done using a GOSlim viewer provided by AgBase (http://agbase.arizona.edu/cgi-bin/tools). 

In order to find genes encoding transcription factors (TFs) and other gene expression regulatory proteins, the protein sequences for all root DEGs were retrieved from the EnsemblPlant database (version Hv_IBSC_PGSB_v2) and analyzed using the iTAK (plant transcription factor and protein kinase identifier and classifier) program [70]. The analysis of TFs with significantly enriched putative targets was performed using the TF enrichment tool from the Plant Transcriptional Regulatory Map (http://plantregmap.cbi.pku.edu.cn/tf_enrichment.php) and a “motif” method with threshold *p*-value ≤0.05 was applied to find possible regulatory interactions between TFs and their putative targets. All Venn diagrams were drawn using Venny 2.1 [71].

### 4.5. Quantitative Reverse Transcription (RT)-qPCR

One microgram of total RNA was subjected to DNase treatment and subsequent cDNA synthesis using RevertAid First Strand cDNA Synthesis Kit (Thermo Fisher Scientific, Waltham, MA USA) according to the manufacturer’s instructions. The cDNA was diluted 1:5 with ddH_2_O and used as a template for the qPCR. The primers that were used in the qPCR were designed using Quant-Prime software (http://www.quantprime.de). The 10 μL qPCR reaction contained 2 μL of cDNA, 1 μL of the primer pair mixture (5 μM), and 5 μL of 2× Master Mix (LightCycler 480 SYBR Green I Master; Roche, Indianapolis, Indiana, USA). The qPCR protocol for the amplification on LightCycler 480 real-time PCR instrument (Roche) using the SYBR Green I method was as follows: initial denaturation for 10 min at 95 °C, followed by 45 cycles of 10 s at 95 °C, 15 s at 60 °C and 10 s at 72 °C, followed by a melting-curve analysis. The gene for ADP-ribosylation factor 1 was used as a reference [72]. All analyses were done in three biological replicates. Amplification efficiencies were calculated using LinRegPCR [73]. Calculations of the fold change of expression (FC) were made using the Pfaffl method [74]. Statistical significance of expression differences between control and drought treated samples were tested using REST software [75]. Spearman’s rank correlation coefficient was used to statistically compare microarray and qPCR FC data.

## Figures and Tables

**Table 1:** Summary of differentially expressed genes in CamB and Maresi barley genotypes under drought stress in comparison to control conditions.

Genotype	Organ	No. of Probes		No. of Genes with Known Annotations *	
		Down-Regulation	Up-Regulation	Down-Regulation	Up-Regulation
CamB	Roots	792	1019	519	536
CamB	Leaves	597	705	354	429
Maresi	Roots	999	884	634	452
Maresi	Leaves	602	717	353	436

*p* < 0.05; fold change (FC) ≥ 3. * barley high confidence genes.

**Table 2:** The list of DEGs involved in the gene expression regulation processes that were specific to the roots of the drought-tolerant CamB genotype.

Horvu ID	Expression Change				Family Name	Gene Description
	CamB Roots	CamB Leaves	Mar Roots	Mar Leaves		
**Transcription factors:**						
HORVU4HR1G087580	up	n/c	n/c	n/c	bHLH	Uncharacterized
HORVU3Hr1G073470	up	n/c	n/c	n/c	CPP	Protein tesmin/TSO1-like CXC 5
HORVU2Hr1G020140	up	n/c	n/c	up	GARP-G2-like	Two-component response regulator ARR18
HORVU7Hr1G096430	down	n/c	n/c	n/c	GARP-G2-like	Myb-like transcription factor family protein
HORVU5Hr1G110960	up	n/c	n/c	n/c	General TF	Transcription initiation factor IIE subunit beta
HORVU1Hr1G071620	up	n/c	n/c	n/c	NF-YB	Nuclear transcription factor Y subunit B
HORVU5Hr1G062040	up	n/c	n/c	n/c	SCAI TF family	Protein SCAI
HORVU3Hr1G061930	down	down	n/c	down	TUB	Tubby-like F-box protein 1
**Transcriptional Regulators:**						
HORVU5Hr1G061120	down	n/c	n/c	n/c	BAH domain	Bromo-adjacent homology (BAH) domain-containing protein
HORVU6Hr1G034680	up	n/c	n/c	n/c	PHD	methyl-CPG-binding domain 9

n/c—no change.

**Table 3:** The list of DEGs involved in the gene expression regulation processes that were specific to the roots of the drought susceptible Maresi genotype.

Horvu ID	Expression change				Family Name	Gene Description
	CamB Roots	CamB Leaves	Mar Roots	Mar Leaves		
**Transcription factors:**						
HORVU2Hr1G050260	n/c	n/c	up	n/c	AP2/ERF-ERF	Ethylene-responsive transcription factor
HORVU4Hr1G077310	n/c	n/c	down	n/c	AP2/ERF-ERF	Ethylene-responsive transcription factor
HORVU5Hr1G080300	n/c	n/c	down	down	AP2/ERF-ERF	Dehydration-responsive element-binding protein 1B
HORVU6Hr1G080340	n/c	up	up	up	AP2/ERF-ERF	Ethylene-responsive transcription factor 5
HORVU3Hr1G105720	n/c	n/c	down	n/c	B3	B3 domain-containing protein
HORVU5Hr1G017450	n/c	n/c	up	n/c	B3	B3 domain-containing protein
HORVU3Hr1G108680	n/c	n/c	up	n/c	bHLH	Transcription factor ORG2
HORVU5Hr1G002090	n/c	n/c	down	n/c	bHLH	Basic helix-loop-helix (bHLH) DNA-binding superfamily protein
HORVU6Hr1G071950	n/c	n/c	down	n/c	C2C2-CO-like	Zinc finger protein CONSTANS-LIKE 10
HORVU4Hr1G088280	n/c	up	up	up	C2C2-GATA	GATA transcription factor 2
HORVU3Hr1G085720	n/c	n/c	down	n/c	HB-BELL	BEL1-like homeodomain 8
HORVU5Hr1G067010	n/c	n/c	down	n/c	HB-HD-ZIP	Homeobox-leucine zipper protein family
HORVU2Hr1G061320	n/c	n/c	down	n/c	HB-KNOX	Homeobox protein knotted-1-like 12
HORVU5Hr1G098570	n/c	n/c	down	n/c	HB-KNOX	Homeobox protein knotted-1-like 12
HORVU0Hr1G003020	n/c	n/c	down	n/c	MADS-M-type	MADS-box transcription factor 18
HORVU3Hr1G095090	n/c	n/c	down	n/c	MADS-M-type	MADS-box transcription factor family protein
HORVU3Hr1G052710	n/c	n/c	down	n/c	MYB-related	Myb-like transcription factor family protein
HORVU6Hr1G066000	n/c	down	down	n/c	MYB-related	Myb-like transcription factor family protein
HORVU5Hr1G045640	n/c	n/c	down	down	NAC	NAC domain protein,
HORVU2Hr1G072420	n/c	n/c	down	n/c	S1Fa-like	DNA-binding protein S1FA2
HORVU3Hr1G094730	n/c	n/c	down	n/c	SBP	Squamosa promoter-binding-like protein 2
HORVU1Hr1G054620	n/c	n/c	down	n/c	STAT	SH2 domain protein B
HORVU3Hr1G095400	n/c	n/c	down	down	TCP	TCP family transcription factor
HORVU6Hr1G093960	n/c	n/c	down	n/c	TCP	TCP family transcription factor
HORVU7Hr1G038130	n/c	n/c	down	n/c	TCP	TCP family transcription factor
HORVU7Hr1G041230	n/c	n/c	down	up	Tify	Protein TIFY 3A
HORVU1Hr1G069620	n/c	n/c	down	n/c	WRKY	WRKY DNA-binding protein 54
HORVU3Hr1G050590	n/c	n/c	down	n/c	WRKY	WRKY DNA-binding protein 50
**Transcriptional regulators:**						
HORVU6Hr1G091700	n/c	n/c	down	n/c	Others	Ethylene receptor 3
HORVU2Hr1G017680	n/c	up	down	up	PHD	E3 ubiquitin-protein ligase SHPRH
HORVU4Hr1G003040	n/c	n/c	down	n/c	TRAF	Regulatory protein (NPR1)

n/c—no change.

**Table 4:** The transcription factors that possess significantly over-represented targets within root DEGs.

Horvu ID	MLOC ID	Background-All ^1^	Background-Bind ^2^	Query-All ^3^	Query-Bind ^4^	*p*-Value	*q*-Value	Description (IPK)
HORVU1Hr1G090030	MLOC_6711	24306	470	546	21	1.059 × 10^−3^	3.352 × 10^−2^	G-box binding factor 2
HORVU5Hr1G014170	MLOC_81003	24306	388	546	16	7.159 × 10^−3^	9.715 × 10^−2^	Basic-leucine zipper (bZIP) TF family protein
HORVU7Hr1G056820	MLOC_75166	24306	368	546	15	9.549 × 10^−3^	1.210 × 10^−1^	Heat stress transcription factor B-2b
HORVU6Hr1G068100	MLOC_14844	24306	386	546	14	2.906 × 10^−2^	2.301 × 10^−1^	Myb-like TF family protein

^1^—no. of reference genes in the database; ^2^—no. of genes from reference list that may be the targets of TFs; ^3^—no. of genes in the query list; ^4^—no. of genes from the query list that may be the targets of TFs; *p*-value, *q*-value—the significance of the enrichment analysis without and with false discovery rate adjustment, respectively.

**Table 5:** Genes that show an opposite expression pattern under mild and severe drought stress.

Horvu ID	MLOC ID	Family Name	Mild Drought				Severe Drought				Description
			Roots		Leaves		Roots		Leaves		
			CamB	Mar	CamB	Mar	CamB	Mar	CamB	Mar	
HORVU7Hr1G089930	MLOC_59305	AP2/ERF-ERF	up	up	n/c	n/c	down	down	n/c	n/c	Ethylene-responsive transcription factor 1
HORVU5Hr1G047610	MLOC_5148	LOB	up	up	n/c	n/c	n/c	down	n/c	n/c	LOB domain-containing protein 4
HORVU2Hr1G017460	MLOC_65101	NAC	up	up	n/c	up	down	down		up	NAC domain protein
HORVU1Hr1G081570	MLOC_32433	WRKY	up	up	n/c	n/c	down	n/c	n/c	n/c	WRKY DNA-binding protein 24
HORVU3Hr1G108680	MLOC_36351	bHLH	n/c	up	n/c	n/c	down	down	n/c	n/c	Transcription factor ORG2

n/c—no change.

## Supplementary Materials

Supplementary materials can be found at https://www.mdpi.com/1422-0067/20/24/6139/s1.

## Abbreviations

DEG	Differentially expressed gene
GO	Gene ontology
PSI	Photosystem I
PSII	Photosystem II
ROS	Reactive oxygen species
TF	Transcription factor
