# Supplementary material for: Insights into Barley Root Transcriptome under Mild Drought Stress with an Emphasis on Gene Expression Regulatory Mechanisms
Source: Int J Mol Sci. 2019 Dec 5;20(24):6139. doi: 10.3390/ijms20246139 (PMC6940957; doi:10.3390/ijms20246139)
Supplement: Supplementary file 1 [file ijms-20-06139-s001.zip › Supplementary Figures.pdf]

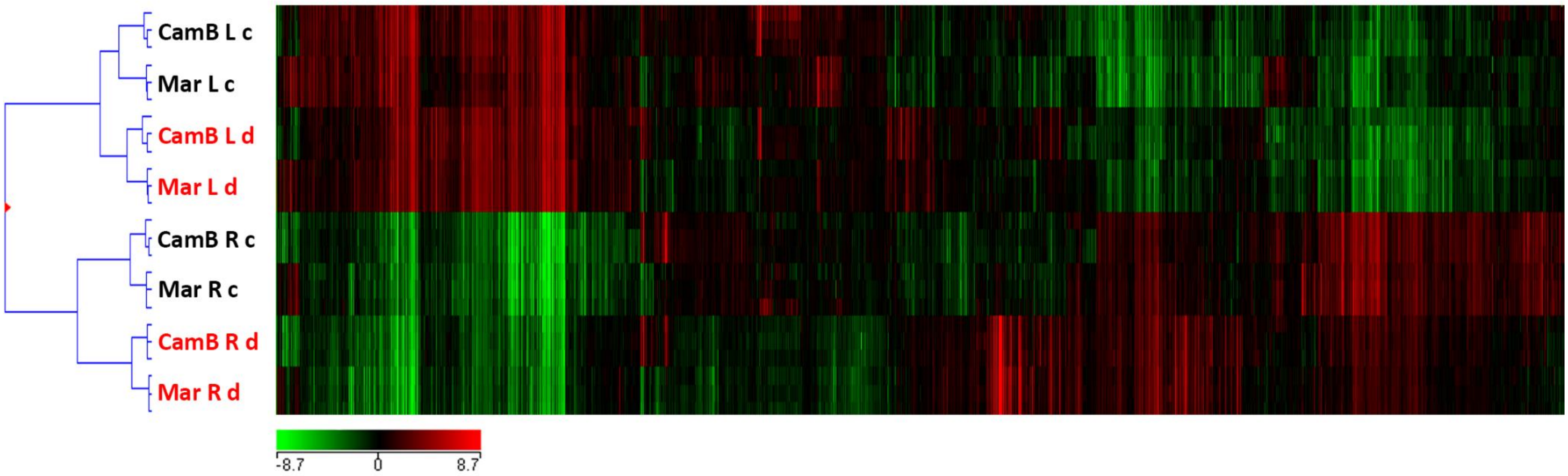

Supplementary Figure S1. Hierarchical clustering of microarray data from CamB and Maresi (Mar) genotypes, L – leaves, R – roots, c – control conditions, d – drought stress

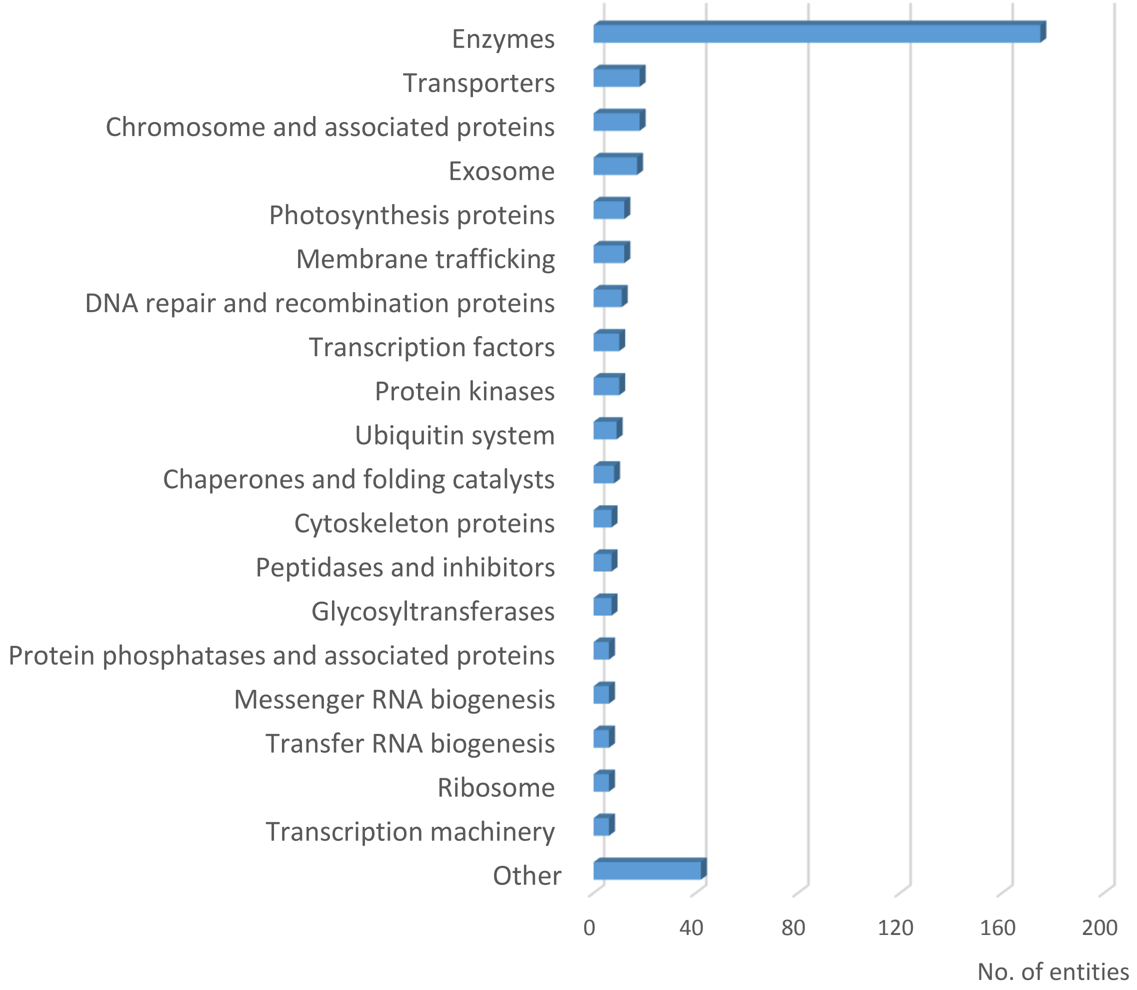

Supplementary Figure S2. BRITE functional hierarchies of all genes differentially expressed upon mild drought treatment of barley seedlings

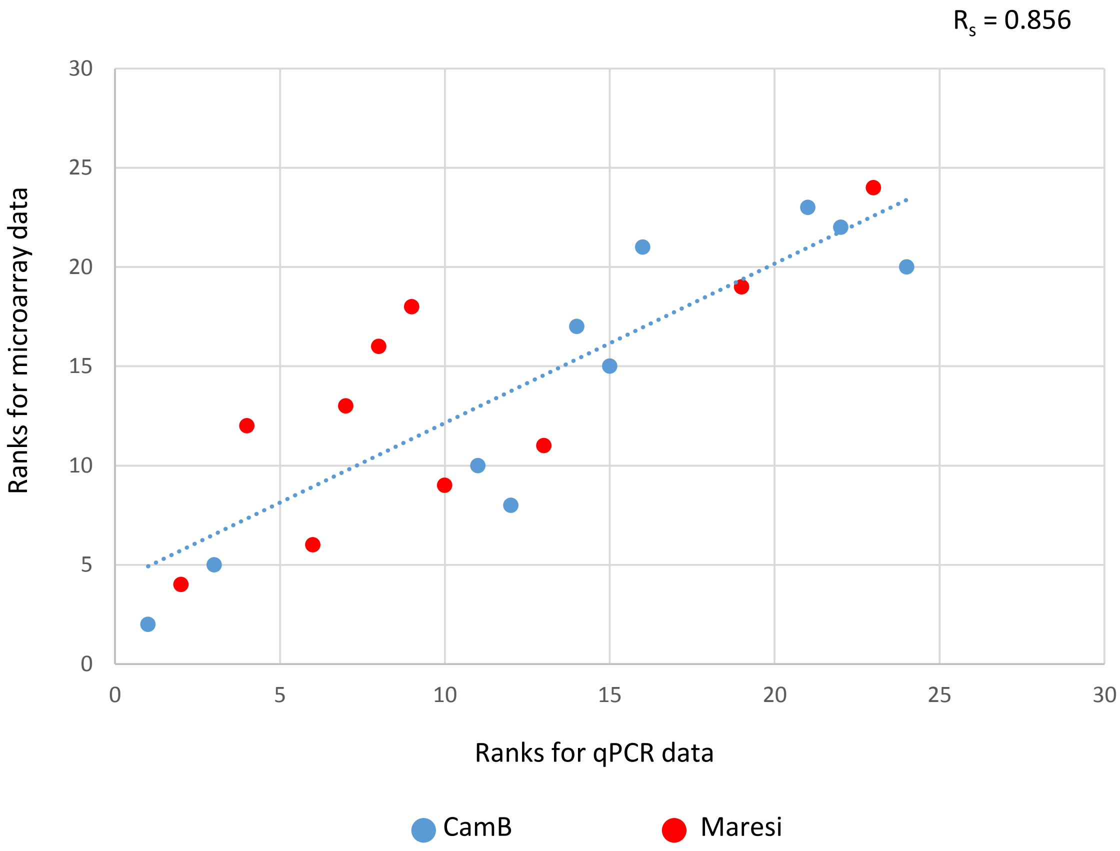

Supplementary Figure S3. Spearman's rank correlation coefficient analysis comparing microarray and qPCR fold change data for selected root DEGs

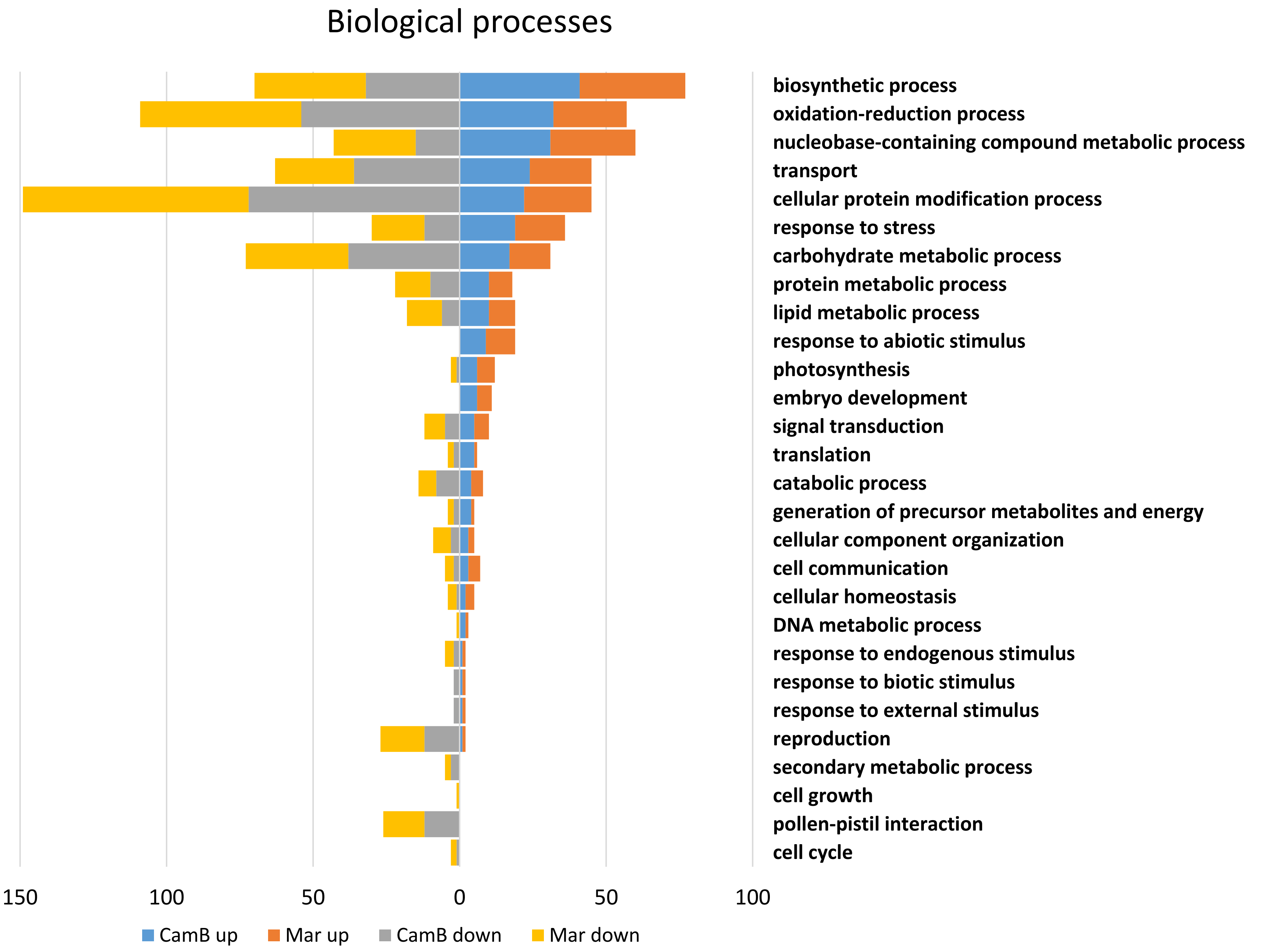

Supplementary Figure S4A. Enrichment analysis of Gene Ontology of Biological processes (GO slim) of genes differentially expressed in roots of CamB and Maresi (Mar) genotypes after the mild drought stress.

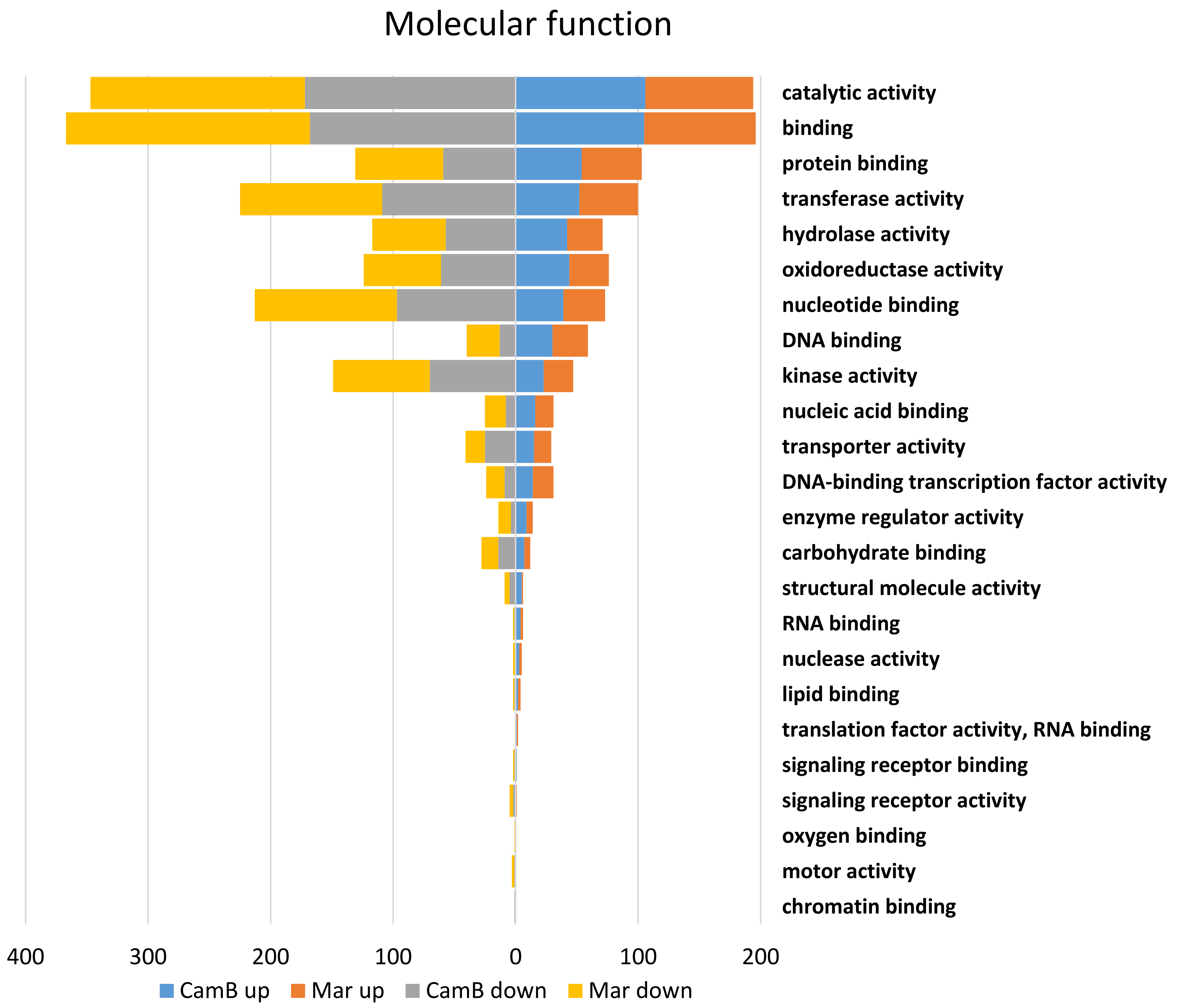

Supplementary Figure S4B. Enrichment analysis of Gene Ontology of Molecular function (GO slim) of genes differentially expressed in roots of CamB and Maresi (Mar) genotypes after the mild drought stress.

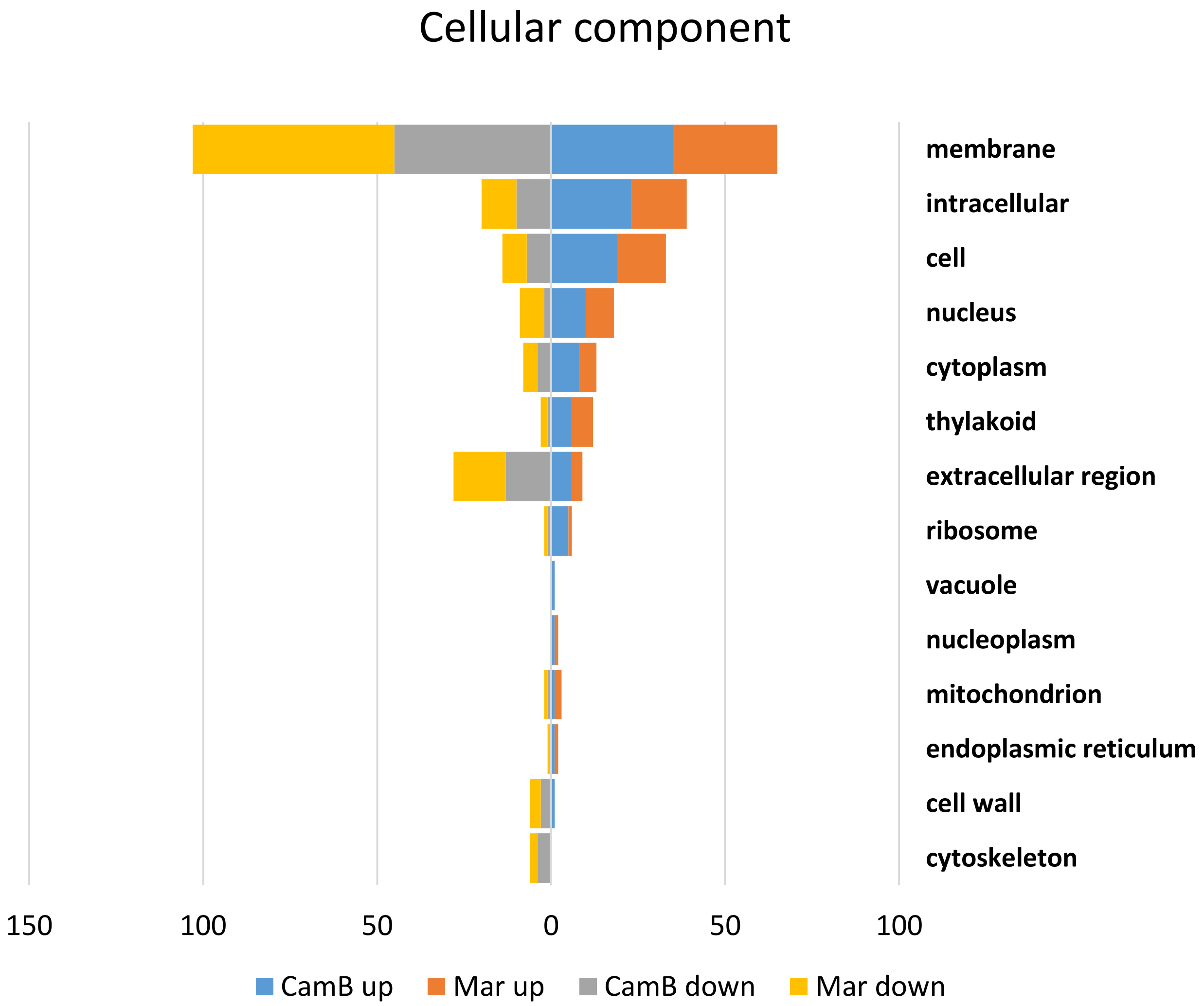

Supplementary Figure S4C. Enrichment analysis of Gene Ontology of Cellular component (GO slim) of genes differentially expressed in roots of CamB and Maresi (Mar) genotypes after the mild drought stress.
